# Effects of Warm-Ups with Weighted Vests and Resistance Bands on Physical Fitness and Combat Ability of Kumite Karate Athletes

**DOI:** 10.3390/sports12030079

**Published:** 2024-03-12

**Authors:** Christos Ioannides, Chrysanthi Despotopoulou, Marios Hadjicharalambous, Nikolaos Zaras

**Affiliations:** 1 Human Performance Laboratory, Department of Life Sciences, School of Life and Health Sciences, University of Nicosia, Nicosia 2417, Cyprus; christos.ioannides2912@gmail.com (C.I.); x.despotopoulou@gmail.com (C.D.); hadjicharalambous.m@unic.ac.cy (M.H.); 2 Department of Physical Education and Sport Science, Democritus University of Thrace, 69100 Komotini, Greece

**Keywords:** kumite, post-activation performance enhancement, combat ability, reaction time

## Abstract

The purpose of this study was to investigate whether a dynamic warm-up either with weighted vests (WVs) or with resistance bands (RBs) may enhance the physical fitness and combat ability of kumite karate athletes. Nine male athletes (age range from 16 to 30 years), participated in this study. Athletes followed three different warm-up programs in a randomized order: (a) with their body mass only (C), (b) with a WV of 10% load of their body mass and (c) with a wearable RB of 10% tension of their body mass. Following the warm-up, athletes performed the following tests: countermovement jump (CMJ), standing long jump (LJ), punch and kick reaction time, bench press throws, T-Half test and the kick frequency test. CMJ remained unaltered following the WV and RB warm-up compared to the C. Standing long jump increased significantly following the RB warm-up compared to the C (2.2 ± 1.7%, *p* = 0.011). Punch reaction time was significantly shortened following the RB warm-up compared to C (−7.3 ± 5.9%, *p* = 0.019). No changes were observed for kick reaction time, bench press throws or the T-Half test. The kick decrement index, measured from the kick frequency test, was significantly decreased following the RB warm-up compared to the C (33.1 ± 20.2%, *p* = 0.011). These results suggest that an RB warm-up may significantly enhance the physical fitness and combat ability in kumite karate athletes.

## 1. Introduction

Karate is a full-contact combat sport that is divided into kata and kumite. Kata consists of several pre-arranged fighting techniques including offensive and defensive actions with an imaginary opponent where technique, rhythm, power, movement flow and kime (holding a powerful position at the end of each technique) are the key criteria for achieving a successful performance [1]. On the other hand, kumite refers to the actual combat between two opponents who, under specific rules, are allowed to use punching and kicking techniques in an attempt to earn points and win the combat [1]. The present study focuses on kumite karate athletes. Kumite is considered to be a high-intensity combat sport, where the aerobic metabolic system provides the majority of the energy contribution during a competition in addition to the ATP-PCr system, which is also a substantial energy provider during fast offensive and defensive muscle actions [2]. Considering that the duration of a punch and a kick ranges between 0.123 and 0.150 s and 0.177 and 0.293 s, respectively [2,3], it is obvious that kumite athletes need to apply their force rapidly, having a high reaction time during offensive and defensive actions and being able to endure the total duration of combat (3 min) at high intensities. Although long-term training may enhance the physical fitness and combat ability of kumite athletes [4,5], coaches may use various warm-up strategies before entering a competition, which may acutely enhance kumite performance.

Warm-up is a training strategy that is applied before training and official competitions, aiming to prepare the athlete for the demanding and high-intensity muscle actions that the sport requires [6]. The warm-up induces several physiological changes, such as an increase in muscle temperature, an increase in blood flow at the periphery, and subsequently, an increase in muscle fiber performance [6,7], mainly due to the presence of the post-activation performance enhancement (PAPE) phenomenon [8,9]. PAPE is a short-lived phenomenon that may acutely enhance athletic performance by increasing the phosphorylation of myosin light chains in the sarcomeres, thus increasing the recruitment of higher threshold motor units and possibly the modification of the fascicle angle of the recruited muscles [10]. These positive effects of a warm-up may lead to a significant increase in performance in several sports [11,12]. As a training strategy, the warm-up may be separated into the following two parts: a general and a specific warm-up. The general warm-up includes low-intensity exercises such as running and static stretching, aiming to increase muscle temperature, flexibility, blood flow and the general readiness of the athlete. On the contrary, the specific warm-up aims to progressively increase the warm-up intensity by adding dynamic stretching and high-intensity sport-specific exercises [13]. However, the effect of a warm-up on the physical fitness and combat ability of kumite athletes remains largely unclear.

During the past few decades, the warm-up with an external resistance load has been a popular training strategy for several sports [14,15,16]. External loads such as weighted vests and resistance bands have been used to acutely increase athletic performance. More specifically, a study on eight elite badminton players examined the effect of a warm-up without an extra load and with 5 and 10% loads of their body mass with weighted vests on the athletes’ vertical jump and ability to change direction. The study showed that the warm-up with weighted vests significantly enhanced their ability to change direction but not their vertical jump compared to the unloaded condition [17]. In line with these results, a study of 19 male soccer players examined the effects of an unloaded warm-up and three different loads of weighted vests including 5, 10 and 15% of their body mass on repeated sprint ability immediately (15 s), 5 and 8 min following the warm-up. The study showed that all three loaded conditions enhanced their repeated change of direction ability [18]. However, a study on 16 high-school football players explored a standard warm-up with body mass and a weighted vest of 5% of the individual players’ body mass. The results of the study showed no significant difference in lower body power output between the weighted vest and unloaded condition [19]. However, the effect of a warm-up with a weighted vest on the physical fitness and combat ability of kumite athletes remains unclear.

In contrast to weighted vests, several studies have focused on the effects of a warm-up with resistance bands. A study on 16 martial arts athletes (from kickboxing and taekwondo) investigated the effects of a warm-up session with either a resistance band or an unloaded condition. The study showed that kicking velocity during the roundhouse kick increased by 3.3% after the resistance band warm-up, with concomitant increases in muscle activation of the vastus medialis and rectus femoris muscles [20]. In addition, a study on 11 male judo athletes investigated the effects of a warm-up including upper and lower body resistance bands and broad jumps, a warm-up including lower body broad jumps and an unloaded warm-up before measuring the athletes’ performance in the special judo fitness test and a high pull. Upper and lower body warm-ups with resistance bands and broad jumps led to a significant increase in power and the number of throws made during the specific judo fitness test compared to the unloaded condition [21]. Although both weighted vests and resistance bands have been used in previous studies, the effects of warm-ups with either a weighted vest or a resistance band on the physical fitness and combat ability of kumite athletes remain unexplored.

Therefore, the aim of this study was to investigate the effects of a warm-up with weighted vests and a warm-up with resistance bands on the physical fitness and combat ability of kumite karate athletes. The hypothesis of this study was that both loaded warm-up strategies would induce significantly higher physical fitness and combat ability increases compared with a warm-up only with body mass.

## 2. Materials and Methods

### 2.1. Experimental Design

The effect of a warm-up with either weighted vests or resistance bands on the physical fitness and combat ability of kumite karate athletes remains unclear. Nine well-trained kumite athletes followed three different warm-up conditions as follows: (a) with their body mass only (control, C), (b) with a weighted vest (WV) loaded with 10% of their individual body mass and (c) with a wearable resistance band (RB) at 10% tension of their individual body mass. All athletes underwent all three conditions randomly. Before the warm-up conditions, the athletes visited the laboratory for an evaluation of their anthropometric characteristics, while on a different day, they visited the karate dojo for a familiarization session including the warm-up routines and the performance measurements. Then, during a 3-day period separated by 96 h, the athletes followed the three different warm-up conditions. Differences among the three conditions were statistically compared.

### 2.2. Participants

Eleven male kumite karate athletes participated in this study. Two of them withdrew from the experimental procedures for reasons unrelated to this study. Consequently, 9 male kumite karate athletes [age: 19.3 ± 5.1 years (age range from 16 to 30 years); body mass: 69.3 ± 12.0 kg; body height: 1.73 ± 0.05 m] with 11.0 ± 4.5 years of competitive experience participated in this study. All the athletes were members of the National karate team and among the top three in their individual body mass category in the Nation, while seven of them had earned podium honors in international Small States competitions. Three of them won 2nd, 5th and 9th place during the last European championship, while one of the athletes won 2nd place during the last Mediterranean games. Athletes were informed about the experimental procedures and signed an informed consent form. For athletes under the age of 18, parental consent was obtained. Before entering this study, athletes fulfilled the following criteria: absence of any cardiovascular issue or muscle injury, training on a regular basis (>8 training sessions per week) and competition in the last National championship. In addition, if an athlete consumed energy supplements (i.e., caffeine), had an injury, performed vigorous exercise the day before measurements or had not eaten properly at least 3 h before entering the measurements, then he was excluded from this study. All procedures were in accordance with the 1975 Declaration of Helsinki as revised in 2000 and were approved by the National Ethics Committee of Cyprus (project number: EEBK/ΕΠ/2022/78).

### 2.3. Warm-Up Conditions

All warm-up conditions were performed in the same karate dojo for all athletes during noon hours (between 13:00 and 16:00 h) at an ambient temperature of 26 °C. All measurements were performed following the European championship and a local competition; consequently, measurements were performed during the competition training phase. The athletes followed all three warm-up conditions in a randomized order. The warm-up program was designed with a progressive increase in intensity and separated into the following 2 levels: the 1st level (general warm-up) included low-intensity running and static stretching as well as dynamic exercises for the whole body with moderate intensity, and the 2nd level (specific warm-up) included ballistic exercises and basic kumite techniques at high intensity. The warm-up exercises were the same for all three conditions. The actual warm-up protocol is presented in Table 1. The warm-up had a duration of approximately 12 min. At the end of the warm-up, an 8-min rest period was allowed for athletes since previous studies have shown that 8 min is a sufficient period of time to enhance performance following a loaded warm-up in athletes [10,18]. At the end of the 8 min of rest, the athletes provided their personal rate of perceived exertion (RPE) on a scale of 6–20. Athletes performed the warm-up either with their own body mass (C), with a WV with an additional loading of 10% of their individual body mass or with a wearable RB, where the bands were fixed to provide a tension equal to 10% of their body mass. In order to measure the tension of the bands, we performed a measurement on the resistance bands using a tension sensor (Applied Measurements Ltd., DBBE-1000 kg, Aldermaston, Berkshire, UK). The band was stretched for a total of 30 cm, and the tension was measured as the force from the sensor for each 1 cm of stretch. Then, the individual resistance tension for each athlete was calculated, and the two loaded warm-up conditions were equalized. According to the results from the body composition analysis, we decided to share the 10% tension from the resistance bands on 3% on their upper body (1.5% on each arm) and 7% on their lower body (3.5% on each leg).

### 2.4. Body Composition Analysis and Familiarization Session

During the first day of this study, the athletes visited the laboratory for the evaluation of their anthropometric characteristics and body composition. Measurements were performed during the morning hours, and the athletes were instructed to fast for approximately 8 h and abstain from any strenuous exercise during the previous day [22]. The body composition analysis was performed on a bioelectrical impedance scale (Tanita MC-780MA, Tokyo, Japan), and included the evaluation of fat-free mass and percentage body fat. Briefly, the body height of the athletes was initially measured with a measuring tape, which was positioned on the laboratory wall. Then, the athletes stepped on the bioelectrical impedance scale with light clothing and without shoes or socks and stood still for approximately 45 s. The intra-class correlation coefficient (ICC) for body mass, percentage body fat and fat-free mass were: 0.998 (95% CI: lower = 0.998, upper = 0.999), 0.990 (95% CI: lower = 0.982, upper = 0.995) and 0.965 (95% CI: lower = 0.958, upper = 0.987), respectively.

During the second day of this study, the athletes visited the karate dojo for the familiarization session including the warm-up procedure and the performance measurements. Athletes performed the warm-up condition with lower intensity and familiarized themselves with all the performance measurements. In addition, at the end of the familiarization session, the one repetition maximum strength (1-RM) test in the bench press was performed. More specifically, 1-RM strength was measured on a Smith machine while a full range of motion was used, with the barbell lowered until the chest and back up. The athletes performed multiple sets with an empty barbell and then performed 8 repetitions at 50% of the predicted 1-RM. Subsequently, the athletes performed 6, 4 and 2 repetitions at 70, 80 and 90% of their predicted 1-RM, respectively. Thereafter, the athletes had 3 attempts for the determination of 1-RM strength with 3 min of rest between attempts [23]. During all repetitions, the athletes were instructed to lift the loads with maximum velocity during the concentric phase of movement, regardless of the actual movement velocity [24]. The ICCs for 1-RM strength in the bench press was 0.966 (95% CI: lower = 0.914, upper = 0.985). Following the 1-RM strength test, the athletes performed a familiarization bench press throw test, which included 4 sets of 5 repetitions/throws at 30% of 1-RM.

### 2.5. Countermovement Jumps

The evaluation of the CMJ with arms akimbo was performed first following the warm-up conditions (OptoJump Next, Microgate, Bolzano, Italy). Briefly, 8 min after the end of each warm-up condition, 3 maximum CMJs were performed with arms akimbo. During the CMJs, the athletes were instructed to jump as high as possible, and 30 s of rest was allowed between jumps. Following each jumping trial, the researchers informed the athletes about the jumping height in order to motivate them to expend greater effort. The mean performance of all CMJs was used for the statistical analysis. From each CMJ, the jumping height, power and power relative to body mass were evaluated. The ICC for CMJ height, power and power per body mass were 0.989 (95% CI: lower = 0.957, upper = 0.997), 0.980 (95% CI: lower = 0.985, upper = 0.990), and 0.981 (95% CI: lower = 0.978, upper = 0.991), respectively. Following the CMJ with arms akimbo, the CMJ with arm swing (CMJas) was performed. Similarly, 3 maximum attempts were allowed with 30 s of rest. Similar to CMJs, the athletes had the same instructions as to jump as high as possible, and they were given feedback regarding the jumping height of each jump. The mean value of all three CMJas was used for the statistical analysis. The ICC for CMJas height was 0.92 (95% CI, upper = 0.98, lower = 0.89).

### 2.6. Standing Long Jump

One minute after the CMJas, the standing long jump was performed. Briefly, the athletes placed their toes at the beginning of the measuring tape in front of a mattress in an attempt to push with their lower body and jump with arm swing as long as possible [5]. The distance of each long jump was measured to the nearest centimeter from the take-off point to the mark where the heels landed on the mattress. In addition, marks were placed on the mattress in order to motivate the athletes to jump even further. Three maximum standing long jumps were performed with 30 s of rest between attempts. The mean value of all standing long jump attempts was used for the statistical analysis. The ICC for the standing long jump was 0.961 (95% CI: lower = 0.884, upper = 0.987).

### 2.7. Punch and Kick Reaction Time

Two minutes after the standing long jump test, the athletes performed the reaction time test. Reaction time was evaluated with the blazepod system (Blazepod, Miami, FL, USA). Punches were first evaluated with the kiakou-zouki technique and afterward, kicks followed with the kiakou-mawashi geri technique. For punches, one pod was placed on a stand bag at a height equal to the height of the center of the sternum of each athlete, while for kicks, the pod was placed on the side of the stand bag equal to the lower ribs of each athlete. The athletes were placed at a self-selected distance from the stand bag which was measured as the distance between the front foot and the stand bag base for both punches and kicks. The same distance was used following all warm-up conditions. The athletes were instructed to maintain their technique constant during all hits. Then, the athletes had 5 attempts to hit the pod as fast as possible. Only a red visual signal was given to the athletes by the pod, while the first attempt was deleted from the analysis because the pod light turned on automatically at the beginning of the test. The pod was set to light up randomly between 3 and 7 s for the following 4 attempts. During all attempts, the athletes were vocally informed about the reaction time and encouraged to be faster. The mean value of the 4 attempts for both the punches and kicks was used in the statistical analysis. The ICC for the reaction time test was 0.965 (95%CI: lower = 0.940 upper = 0.995).

### 2.8. Bench Press Throws

Immediately after the end of the reaction time test, the athletes performed bench press throws with 30% of their individual 1-RM [25,26]. The athletes laid on the bench of the Smith machine and lowered the barbell to their chest where the load was placed on the machine breaks. Then, the athletes were instructed to throw the barbell as high as possible. One researcher was present to catch the barbell at the highest point [26]. Only concentric muscle action was allowed and was measured via a linear encoder (Vitruve encoder, Vitruve fit, Madrid, Spain). The athletes performed 3 maximum bench press throws with 10 s of rest between throws. After each throw, the athletes were informed about the mean propulsive velocity and encouraged to throw the barbell even faster. The mean value of all three bench press throw attempts was used in the statistical analysis, whilst the mean propulsive velocity, the peak velocity and power were measured from the linear encoder. The ICC for the bench press throw was 0.850 (95%CI: lower = 0.755, upper = 0.912).

### 2.9. T-Half Agility Test

One minute after the bench press throws, the athletes performed the T-Half test [27]. The athletes had 2 maximal attempts with 2 min of rest. One photocell gate (Witty, Microgate, Bolzano, Italy) was positioned at the starting line approximately 1 m above the ground and 3 m apart, while the athletes initiated their sprinting effort 0.4 m behind the starting line. For an athlete who failed to maintain a facing forward position, crossed his feet or failed to touch a cone during an attempt, then, this attempt immediately stopped, and the athlete was given an extra attempt. The mean time-trial performance from both attempts was used for the statistical analysis. The ICC for the T-Half test was 0.94 (95% CI: lower = 0.82, upper = 0.96).

### 2.10. Frequency Kick Test

Two minutes following the T-Half test, the athletes performed the frequency kick test on a stand bag. The frequency kick test included repeated kicks with alternating legs using the kiakou-mawashi geri technique. The athletes performed repeated kicks for 5 sets of 10 s with 10 s of rest between sets (1:1 exercise-to-rest ratio). During the test, the athletes tried to accomplish as many kicks as possible [28]. The height of the kicks on the stand bag was individualized according to the height of each athlete. More specifically, an electronic Taekwondo chest protector was placed on the stand bag equally to the chest height of each athlete. The athletes were instructed to kick at the sides of the chest protector in order to measure the hit. From the frequency kick test, the number of kicks during all sets, the maximum number of kicks during one set and the kick decrement index (KDI) [29] were measured. The ICC for the frequency kick test was ICC = 0.854 (95%CI: lower = 0.785 upper = 0.958).

### 2.11. Statistical Analysis

All data are presented as mean ± SD and were normally distributed according to the Shapiro–Wilk test. A post hoc sample power analysis showed a power of 0.723 for the total number of athletes in all three groups [30]. A 3-way repeated measures analysis of variance was used to examine differences between the C, WV and RB warm-up conditions. Eta squared (η^2^) was calculated as the effect size for the comparison between conditions. A Bonferroni adjustment was used to compare the main effects between the three warm-up conditions. Hedge’s *g* effect size was also calculated for the comparison between pairs of conditions with the following criteria used to infer the magnitude of the difference: <0.2 (trivial), 0.2–0.5 (small), 0.5–0.8 (moderate) and >0.8 (large) [31]. The reliability of all measurements was determined with a 2-way random effect intra-class correlation coefficient and confidence intervals. Significance was set at *p* ≤ 0.05.

## 3. Results

All athletes completed the experimental procedures without experiencing any injuries. The results from the RPE evaluation showed significant differences among the conditions (*p* = 0.001, η^2^ = 0.889). More specifically, a significant difference was found between the WV and C (WV: 12.1 ± 1.36 vs. C: 9.4 ± 1.8, *p* = 0.004, *g* = 1.207) and between the RB and C (RB: 12.6 ± 1.3 vs. C: 9.4 ± 1.8, *p* = 0.004, *g* = 1.420). No difference was found between the WV and RB for RPE (*p* = 0.998, *g* = 0.239). The body composition analysis showed that the athletes had 57.0 ± 8.0 kg of fat-free mass and 14.9 ± 1.8% of body fat. Table 2 presents the results from the CMJ, the CMJas and the standing long jump following all three warm-up conditions. No significant differences were found in the CMJ and the CMJas among the three warm-up conditions. However, the RB warm-up induced greater increases in the standing long jump compared to the C (2.2 ± 1.7%, *p* = 0.011, *g* = 0.200) but not compared to the WV (1.4 ± 2.7%, *p* = 0.416, *g* = 0.134). No significant difference was found between the WV and C for the standing long jump (0.8 ± 1.9%, *p* = 0.799, *g* = 0.070).

**Figure 1:**
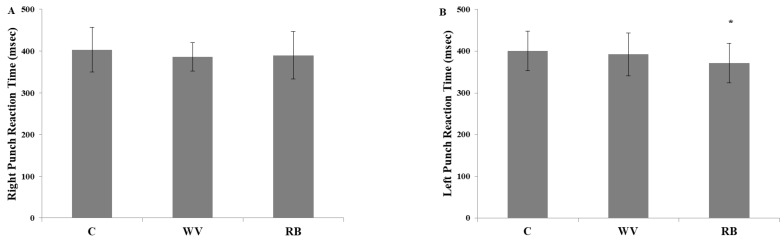
(**A**) Changes in right punch reaction time among conditions. No significant differences. (**B**) Changes in left punch reaction time among conditions. * *p* < 0.05: difference between RB compared with C. C = control, WV = weighted vest, RB = resistance band.

The punch reaction time remained unchanged for the right punch (*p* = 0.207, η^2^ = 0.362). More specifically, no significant differences were observed between the WV and C (−3.7 ± 5.9%, *p* = 0.233, *g* = 0.278), the RB and C (−3.4 ± 6.2%, *p* = 0.411, *g* = 0.181) or between the WV and RB (0.5 ± 6.0%, *p* = 0.987, *g* = 0.054) (Figure 1A). However, significant differences were found (*p* = 0.008, η^2^ = 0.750) in the left punch reaction time between the RB and C (−7.3 ± 5.9%, *p* = 0.019, *g* = 0.450) and almost between the RB and WV (−5.3 ± 5.7%, *p* = 0.058, *g* = 0.313). No difference was observed between the WV and C (−1.8 ± 7.9%, *p* = 0.981, *g* = 0.118) (Figure 1B). Furthermore, no significant differences were found (*p* = 0.253, η^2^ = 0.325) in the right kick reaction time between the WV and C (−0.6 ± 3.9%, *p* = 0.985, *g* = 0.071), between the RB and C (−1.8 ± 3.2%, *p* = 0.386, *g* = 0.146) or between the WV and RB (1.3 ± 3.0%, *p* = 0.792, *g* = 0.097). Similarly, no significant differences were found (*p* = 0.093, η^2^ = 0.492) in the left kick reaction time between the WV and C (0.1 ± 2.7%, *p* = 0.999, *g* = 0.007), between the RB and C (−1.7 ± 2.1%, *p* = 0.128, *g* = 0.140) or between the WV and RB (1.8 ± 2.4%, *p* = 0.170, *g* = 0.141).

The 1-RM in bench press showed that the athletes had 66.1 ± 7.4 kg 1-RM strength. No significant changes were found in the mean propulsive velocity, the peak velocity or the power during bench press throw between conditions (Table 3). Moreover, performance in the T-Half test time trial remained unchanged following the three conditions (*p* = 0.063, η^2^ = 0.547). More specifically, no significant differences were found between the WV and C (−1.8 ± 2.1%, *p* = 0.105, *g* = 0.361), between the RB and C (−1.0 ± 3.7%, *p* = 0.988, *g* = 0.168) or between the WV and RB (−0.7 ± 2.4, *p* = 0.995, *g* = 0.124).

The results of the frequency kick test are presented in Table 4. More specifically, no significant differences were found for the number of kicks during all sets among conditions. However, a significant difference was found for the KDI (*p* = 0.011, η^2^ = 0.722). More specifically, the RB warm-up induced significantly lower KDI compared to the C (RB: −6.54 ± 1.81% vs. C: −10.54 ± 3.8%, *p* = 0.011, *g* = 0.963). No significant difference was found between the WV and C (WV: −8.98 ± 2.33% vs. C: −10.54 ± 3.8%, *p* = 0.992, g = 0.350) or between the WV and RB (*p* = 0.188, g = 0.852), although a large effect size was observed.

## 4. Discussion

The purpose of the current study was to investigate the effects of a warm-up with WVs and RBs on the physical fitness and combat ability of kumite karate athletes. The results of the present study suggest that a warm-up protocol including a wearable RB may lead to a significant increase in the physical fitness and combat ability of kumite karate athletes. Therefore, the hypothesis of this study was confirmed mainly for the RB warm-up condition. Although no significant differences were observed between the WV compared to the C, the warm-up with the WV maintained the physical fitness and combat ability similar to the C. Consequently, coaches may choose a warm-up with RBs before training or competition in an attempt to increase their athlete’s physical fitness and combat ability. However, weighted vests should not be avoided, and coaches may assess their usefulness first under training conditions and then in competition.

A warm-up either with a WV or an RB induced significantly greater RPE values compared to the C warm-up. Athletes completed the same warm-up protocol but with higher external loads compared to the C condition. Both the WV and RB had a load equal to 10% of the individual body mass of each athlete, while no difference was found in RPE between the WV and RB. A study on judo athletes found lower RPE values following resistance band warm-up compared to the C, but the resistance band warm-up had lower training volume compared to the C condition [21]. It is suggested that the warm-up routine should be less tiring for athletes in order to preserve their energy reserves for training or for competition [32]. The higher external load from the WV and RB warm-ups may lead to higher RPE values, and coaches should take this into consideration when they design warm-up protocols before training or competitions.

The vertical jump performance as measured in the current study with the CMJs remained unchanged following the WV and RB warm-ups. A possible explanation for this finding might be the potential insufficient rest period between the end of the warm-up and the initiation of measurements. However, a previous meta-analysis showed that athletes may benefit from the positive effects of PAPE approximately 5 to 7 min after conditioning [10]. Moreover, a study on soccer players showed that 8 min of rest following the warm-up may induce significant increases in the change of direction performance [18], while a study on martial arts athletes (kickboxing and taekwondo) showed that 5 to 8 min of rest following a conditioning activity may increase kick velocity [20]. The athletes who participated in the current study were among the best in the Nation; consequently, 8 min of rest might be a sufficient time of rest to reduce fatigue, maintain muscle temperature [7] and benefit from the PAPE phenomenon. Moreover, a study on elite badminton players found no significant changes in the CMJ following a WV warm-up [17], which is in line with a previous study on resistance-trained participants, where a warm-up with four different WVs with different loads failed to induce significant increases in the CMJ [33]. Moreover, in the current study, both the WV and RB warm-ups maintained performance in the CMJs compared to the C. Consequently, coaches may consider using both warm-up conditions without compromising vertical jump performance.

On the contrary, the standing long jump was significantly increased following the RB warm-up compared to the C. It has been previously suggested that in combat sports, like kumite, horizontal movements may be more important compared to vertical movements [5]. During a kumite combat, athletes perform various front, backward, and side movements in an attempt to gain a better fighting position against the opponent. In this sense, a warm-up with the RB may be a useful training strategy for coaches and athletes prior to a kumite training or competition. The WV warm-up maintained the standing long jump performance similar to the C, but no significant difference was found in the RB. A study on collegiate volleyball players showed that a warm-up with a weighted vest may increase standing long jump performance; however, that study was performed on female team sport players [16]. Consequently, when the aim of the warm-up is to increase standing long jump performance, the RB should be preferred.

An interesting finding of the current study was that the RB significantly shortened the punching reaction time compared to the C and almost compared to the WV (*p* = 0.058). According to the authors’ knowledge, this is the first study that examined the effects of a loaded warm-up condition on the combat ability (reaction time) [34] of kumite athletes; consequently, these findings should be interpreted with caution. Reaction time is a key factor for success in combat sports, especially in kumite, since it shows how fast an athlete can respond to an external fighting stimulus coming from an opponent [34]. Kumite includes several rapid techniques that require fast thinking and even faster reaction from an athlete. Indeed, high-level karate athletes have been shown to have faster reaction time than their novice counterparts, which underpins the importance of reaction time in kumite [34]. However, no difference was found in kick reaction time among the conditions, although a trend was observed for the left kick reaction time (*p* = 0.093). Coaches may use both warm-up techniques to maintain lower-body reaction time; however, the RB seems to be more effective for upper-body reaction time. Still, the effect of a warm-up on reaction time in kumite athletes needs further investigation.

No significant changes were found for the variables of the bench press throw test following the warm-up conditions. One possible explanation for this finding was that athletes were not performing systematic resistance training. This is evident from the low 1-RM strength exhibited in the measurements of the bench press, which are relatively lower compared with the findings of a similar study [25]. Generally, martial arts athletes are trained to move/throw their lower and upper limbs as fast as possible (low-load—fast movement velocity) during combat. Consequently, moving higher loads even with 30% of 1-RM may be a higher loading condition, especially for the upper body. In addition, no significant differences were found in the T-Half test, although a trend was observed between conditions (*p* = 0.063), with the WV tending to enhance more the change of direction compared to the C. In contrast, a study on soccer players found that a warm-up with weighted vests ranging from 5 to 10 to 15% of body mass, may equally increase the change in direction in a 20 m repeated sprint test [18]. However, in our study, the modified T-Half test was used [27], which is more suitable for combat athletes. Coaches may consider using both a WV and an RB during warm-up for maintaining and perhaps enhancing change in direction.

One of the main findings of this study was that the RB induced a significant enhancement in the KDI compared to the C and almost compared to the WV (*g* = 0.852). This is the first study to suggest that the KDI may be enhanced following an RB warm-up in kumite karate athletes; consequently, more research is required to reach certain conclusions. The frequency kick test is a high-intensity performance test that mainly involves the lower body, whilst it is also a good assessment test for the evaluation of the athlete’s anaerobic capacity [29]. When the athletes performed the RB warm-up condition, the resistance tension was placed directly on the upper and lower limbs, while during the WV warm-up, the resistance load was placed on the center of the body mass, creating a total body hypergravity condition [35]. It could be hypothesized that the combat ability, as measured here with the reaction time and the KDI, may be enhanced through the RB due to the loading place of the wearable bands on the athletes’ lower and upper limbs, which might induce a loading specificity. Although this hypothesis seems logical, it needs further investigation.

The current study has some limitations. A constant loading of 10% of individual body mass was applied in both the WV and RB conditions. More studies, with different loading conditions in karate athletes are required. The small sample size may limit the generalization of the results. The absence of electromyography evaluation and muscle temperature measurement following the warm-up protocols may have provided useful insights into the nature of the results. In addition, the inclusion of only male kumite athletes may limit the generalization of the results in female kumite athletes. Future studies should focus on female kumite athletes and on the role of warm-up on physical fitness and combat ability. However, the participants of this study were well-trained athletes with national and international distinctions. Moreover, the mean value of all measurements was used in the statistical analysis in order to obtain a better view of the neuromuscular status of the athletes following the warm-up conditions [36]. These positive effects of the warm-up either with the WV or with the RB have a certain duration that remains to be elucidated.

## 5. Conclusions

In conclusion, an RB warm-up with a resistance tension equal to 10% of the body mass may lead to a significant increase in the standing long jump, shortened punch reaction time and reduced KDI compared to a warm-up with body mass. A warm-up with the WV may contribute to maintaining the physical fitness of kumite athletes; however, T-Half test performance might be enhanced. From a practical point of view, the warm-up protocol used in the current study significantly enhanced the physical fitness of the athletes. Thus, regardless of the external loading conditions, coaches may use the particular warm-up program as a guide for preparing kumite karate athletes before training and competitions. Coaches and athletes may use the RB warm-up to enhance their physical fitness and combat ability. Likewise, the warm-up with a WV maintained performance and perhaps may induce increases in change of direction. However, coaches should be aware that the application of a wearable RB or WV should first be performed under training conditions and then during official competitions. Both loading warm-up programs will induce significantly higher metabolic demands, and this should be monitored under training conditions. Wearable RBs and WVs are easy to apply, and coaches may try various loading conditions in an attempt to increase kumite performance.

## Figures and Tables

**Table 1:** Warm-up program for all three conditions.

General Warm-Up (Low to Moderate Intensity)		Specific Warm-Up (Moderate to High Intensity)	
**Running at a slow pace** **(3 min)**	**Standing running with low knees** **(2 sets × 10 s)**	Right front kicks with outstretched leg (10 reps)	Kizami-zouki and kiakou-zouki (10 reps each side)
	15 s rest		
Static stretching for upper and lower body (10 s hold)	Standing running with high knees (2 sets × 10 s)	Left front kicks with outstretched leg (10 reps)	Kizami-mawashi geri jodan (10 reps each side)
	15 s rest		
Alternate lunches front and back (2 sets × 5 reps)	Standing running with heel kicks (2 sets × 10 s)	Right side kicks with outstretched leg (10 reps)	Kiakou-mawashi geri jodan (10 reps each side)
	15 s rest		
Lateral lunches (2 sets × 5 reps)	Jumping jacks (2 sets × 10 s)	Left side kicks with outstretched leg (10 reps)	Kizami-zouki, kiakou-zouki, Kizami-mawashi geri jodan (5 reps each side)
	15 s rest		
	Alternate legs back to front (2 sets × 10 s)	Alternate right and left front kicks with outstretched leg (10 reps)	Kizami-zouki, kiakou-zouki, Kiakou-mawashi geri jodan (5 reps each side)
	15 s rest		
		Alternate right and left side kicks with outstretched leg (10 reps)	Tuck jumps (10 reps)

All kumite techniques were performed with maximum intensity.

**Table 2:** Results from the countermovement jump and the long jump.

	C	WV	RB	*p*	η^2^
CMJ height (cm)	39.3 ± 5.9	40.4 ± 6.3	39.5 ± 5.8	0.425	0.217
CMJ power (w)	3419.7 ± 802.2	3476.8 ± 797.0	3432.1 ± 785.8	0.425	0.217
CMJ power relative to body mass (w·kg^−1^)	48.9 ± 4.6	49.8 ± 4.9	49.2 ± 4.3	0.418	0.221
CMJas height (cm)	46.7 ± 6.0	46.8 ± 6.9	46.9 ± 7.2	0.928	0.021
Standing long jump (m)	2.26 ± 0.17	2.28 ± 0.17	2.32 ± 0.18 *	0.016	0.693

* *p* < 0.05 compared with C, CMJ = countermovement jump, CMJas = countermovement jump with arm swing, C = control, WV = weighted vest, RB = resistance band.

**Table 3:** Results for the mean propulsive velocity, peak velocity and power during the bench press throw.

	C	WV	RB	*p*	η^2^
MPV (m·s^−1^)	0.95 ± 0.12	0.94 ± 0.10	0.97 ± 0.08	0.348	0.260
PV (m·s^−1^)	1.62 ± 0.19	1.59 ± 0.13	1.62 ± 0.11	0.469	0.195
Power (W)	177.4 ± 49.4	177.8 ± 50.2	182.8 ± 50.4	0.325	0.275

MPV = mean propulsive velocity, PV = peak velocity.

**Table 4:** Results of the frequency kick test.

Sets	C	WV	RB	*p*	η^2^
Set 1 (N)	19.1 ± 1.4	19.4 ± 1.4	19.0 ± 1.3	0.748	0.070
Set 2 (N)	18.4 ± 1.7	18.6 ± 1.3	18.6 ± 1.2	0.769	0.072
Set 3 (N)	16.8 ± 1.5	17.8 ± 1.5	17.8 ± 1.5	0.057	0.594
Set 4 (N)	16.1 ± 1.8	16.8 ± 1.3	17.3 ± 1.1	0.097	0.487
Set 5 (N)	16.2 ± 2.3	15.6 ± 1.7	16.8 ± 1.4	0.145	0.424

Frequency kick test was measured as the number of kicks (N). C = control, WV = weighted vest, RB = resistance band.

## Data Availability

The data presented in this study are available on request from the corresponding author.
